# Supplementary material for: Heart-retina time analysis using electrocardiogram-coupled time-resolved dynamic optical coherence tomography
Source: Sci Rep. 2025 Jan 2;15:385. doi: 10.1038/s41598-024-84417-w (PMC11697082; doi:10.1038/s41598-024-84417-w)
Supplement: Supplementary file 4 — Supplementary Material [file 41598_2024_84417_MOESM4_ESM.pdf]

Heart-retina time analysis using electrocardiogram-coupled time-resolved dynamic optical coherence tomography

Supplementary Material

Supplementary Video 1

Electrocardiogram-coupled time-resolved dynamic optical coherence tomography at the optic nerve head. The pulsatile intensity changes at the centre of the arterioles are due to fringe washout.

Supplementary Video 2

Electrocardiogram-coupled time-resolved dynamic optical coherence tomography of an arteriole with synchronised velocity profile and electrocardiogram trace at the optic nerve head. The ocular blood flow pulsations match in frequency with the heart beat.

Supplementary Video 3

Electrocardiogram-coupled time-resolved dynamic optical coherence tomography of three arterioles with synchronised velocity profiles and electrocardiogram trace at the optic nerve head. The ocular blood flow pulsations match in frequency. The arrival time can be calculated and is denoted as the heart-retina time.
